# *Ureaplasma urealyticum, Ureaplasma parvum, Mycoplasma hominis *and *Mycoplasma genitalium *infections and semen quality of infertile men

**DOI:** 10.1186/1471-2334-7-129

**Published:** 2007-11-08

**Authors:** Radhouane Gdoura, Wiem Kchaou, Chiraz Chaari, Abir Znazen, Leila Keskes, Tarek Rebai, Adnane Hammami

**Affiliations:** 1 Department of Microbiology and research Laboratory "Microorganismes et Pathologie Humaine", Habib BOURGUIBA Hospital-Sfax-TUNISIA; 2 Laboratory of Histology-Embryology and Biology of Reproduction, University School of Medicine Sfax TUNISIA

## Abstract

**Background:**

Genital ureaplasmas (*Ureaplasma urealyticum *and *Ureaplasma parvum*) and mycoplasmas (*Mycoplasma genitalium *and *Mycoplasma hominis*) are potentially pathogenic species playing an etiologic role in both genital infections and male infertility. Reports are, however, controversial regarding the effects of these microorganisms infections in the sperm seminological variables. This study aimed at determining the frequency of genital ureplasmas and mycoplasmas in semen specimens collected from infertile men, and at comparing the seminological variables of semen from infected and non-infected men with these microorganisms.

**Methods:**

A total of 120 semen samples collected from infertile men were investigated. Semen specimens were examined by in-house PCR-microtiter plate hybridization assay for the presence of genital ureaplasmas and mycoplasmas DNA. Semen analysis was assessed according to the guidelines of the World Health Organization. Standard parametric techniques (*t*-tests) and nonparametric techniques (Wilcoxon tests) were used for statistical analysis.

**Results:**

The frequency of genital ureaplasmas and mycoplasmas detected in semen samples of infertile men was respectively 19.2% (23/120) and 15.8% (19/120). The frequency of *Ureaplasma urealyticum *(15%) was higher than that of *Mycoplasma hominis *(10.8%), *Ureaplasma parvum *(4.2%) and *Mycoplasma genitalium *(5%). Mixed species of mycoplasmas and ureaplasmas were detected in 6.7% of semen samples.

Comparison of the parameters of the standard semen analysis between the male partners of the infertile couples with and without genital ureaplasmas and mycoplasmas infection showed that the presence of *Mycoplasma hominis *DNA in semen samples is associated with low sperm concentration (*p *= 0.007) and abnormal sperm morphology (*p *= 0.03) and a negative correlation between sperm concentration and the detection of *Mycoplasma genitalium *in semen samples of infertile men (*p *= 0.05). The mean values of seminal volume, pH, vitality, motility and leukocyte count were not significantly related either to the detection of genital mycoplasmas DNA or to the detection of ureaplasmas DNA in semen specimens.

**Conclusion:**

Our results demonstrate that genital mycoplasmas and ureaplasmas seem to be widespread among the male partners of infertile couples in Tunisia. Genital mycoplasmas infections of the male genital tract could negatively influence semen quality. Our results also indicate that PCR-microtiter plate hybridization assay method provides a rapid and effective technique to detect human genital mycoplasmas and ureaplasmas which is useful for etiological and epidemiological studies of these pathogens.

## Background

Mycoplasmas and ureaplasmas, belonging to the family *Mycoplasmataceae *and *Mollicutes *class, are widely distributed in humans, mammals, birds, reptiles, fish, and other vertebrates as well as in plants [1,2]. The genital mycoplasmas represent a complex and unique group of microorganisms that have been associated with a wide array of infectious diseases in adults and infants. The lack of conclusive knowledge regarding the pathogenic potential of *Mycoplasma *and *Ureaplasma spp*. in many conditions is due to a general unfamiliarity of physicians and microbiology laboratories with their fastidious growth requirements which makes it difficult to detect them. We also note their high frequency in healthy persons and the poor design of research studies attempting to deepen the association with the disease as regards the mere presence of the organisms in the lower urogenital tract. Besides, we fail to consider the multifactor aspects of diseases; and thus considering these genital mycoplasmas only as a last resort [3]. The situation is now changing because of a greater appreciation of the genital mycoplasmas as perinatal pathogens and improvements in laboratory detection, particularly with regard to the development of powerful molecular nucleic acid amplification tests [3].*Ureaplasma urealyticum *(*U. urealyticum*), *Mycoplasma hominis *(*M. hominis*)*, Mycoplasma genitalium *(*M. genitalium*) and *Ureaplasma parvum *(*U. parvum*) are thought to be associated with genitourinary infections [4-7].

Genital ureaplasmas (*U. urealyticum *and *U. parvum*) and genital mycoplasmas (*M. genitalium *and *M. hominis*) are natural inhabitants of male urethra contaminating the semen during ejaculation. However, these microorganisms particularly *U. urealyticum *[8-11] are potentially pathogenic species playing an etiologic role in both genital infections and male infertility. During the past decade, evidence for damage caused by *U. urealyticum *to the development and vitality of human embryos has accumulated. In human *in vitro *fertilization systems, the presence of *U. urealyticum *in either semen or female genital tract resulted in a decline in pregnancy rate per embryo transfer [12,13]. The mechanism by which *U. urealyticum *affects sperm quality has not been yet elucidated. Some investigators did not find any correlation between the presence of *U. urealyticum *and semen alteration [11,14]; other works have reported that the presence of *U. urealyticum *in semen was related to a decrease in sperm concentration [8,11,15], in motility [9,15], and/or in morphology [16]. The dual effect of *U. urealyticum *on the sperm activity (inhibition of sperm motility at low pHs and increase of sperm velocity at higher pHs, depending on sperm metabolism) has been recently demonstrated [17].

The impact of *M. hominis *infection on semen parameters and male fertility remains unclear. Hitherto, *M. genitalium *and *U. parvum *have seldom been investigated in semen of infertile men.

Genital mycoplasmas and ureaplasmas infections are commonly diagnosed by culture. However, given the time-consuming culture which requires to 2 to 5 days for *Ureaplasma *spp. and *M. hominis *and up to 8 weeks for *M. genitalium*, infectious agents can be detected in less than 8 hours by nucleic acid amplification techniques. Diagnostic methods for the detection of mycoplasmas and ureaplasmas are not commercially yet available. Recently, a PCR-microtiter plate hybridization assay was developed to detect *M. genitalium, M. hominis, U. parvum *and *U. urealyticum *in urine samples [8]. Thus, we used this PCR-microtiter plate hybridization assay and we tried to detect *U. urealyticum, U. parvum, M. hominis *and *M. genitalium *in semen specimens collected from male partners of infertile couples in Tunisia in order to determine the frequency of these microorganisms. We also compared the seminological variables of semen from men who were infected or colonised with genital ureaplasmas and mycoplasmas and from non-infected men.

Genital mycoplasmas and ureaplasmas seem to be widespread among the male partners of infertile couples in Tunisia and *Mycoplasma hominis *infection of the male genital tract could negatively influence semen quality.

## Methods

### Subjects

A total of 120 men attending obstetrics and gynecology clinics in Sfax (South of Tunisia) for diagnostic semen analysis were selected to the study after they gave informed consent. All men were undergoing semen analysis as part of a work-up for infertility investigations after failing to conceive with their partner after one year of unprotected intercourse. None of the men showed any symptoms of genitourinary infections and were therefore considered asymptomatic of sexually transmitted disease. The mean duration of infertility was 4.9 years (range 1–19). The mean age of patients was 36.9 years (range 26–58). Prior approval by ethic committee (Association d'Enregistrement et de Lutte Contre le Cancer du Sud Tunisien) was obtained.

### Sperm seminological variables

Prior to semen analysis, the men were asked to abstain from sexual intercourse or masturbation for 3–5 days before attending the clinic. All samples for analysis were produced on site and collected into standard containers that had previously been shown not to have any cytotoxic effects on human spermatozoa according to the methods outlined by World Health Organization [18]. Immediately after semen production, samples were placed in an incubator and liquefied at 37°C for up to 30 minutes before analysis. Semen analysis was performed according to the WHO criteria to determine the following variables: seminal volume, pH, sperm concentration, vitality, total progressive motility (category [a + b]), rapid progressive motility (category [a]) and morphology (normal forms). Oligospermia was defined by a sperm concentration < 20 × 10^6^/ml, asthenospermia by a sperm motility <50% (category [a + b]), necrospermia by a sperm vitality < 50% and teratospermia by a normal morphology <30%.

Peroxidase staining, a practical and reliable method recommended by WHO for determining leukocytes in the semen, was employed to count and differentiate leukocytes (white blood cells) from immature germ cells [18]. Leukocytospermia was indicated by a concentration of leukocytes ≥ 10^6^/mL.

### Detection of genital mycoplasmas and ureaplasmas in semen specimens by PCR

For each male patient, 200 μL of semen specimens were used for the detection of genital mycoplasmas and ureaplasmas DNA. An amplification-based method was performed to determine the presence or absence of *U. urealyticum, U. parvum, M. hominis *and *M. genitalium *DNA in these samples according to the following protocol.

### Extraction of DNA by Cetyltrimethylammonium bromide (CTAB)-phenol-chloroform/isoamyl alcohol method

The precipitates from each 200 μL of semen specimens were harvested by centrifugation at 14,000 *g *for 20 minutes. The precipitates were treated with 5 μl of proteinase K (20 mg/ml) at 55°C for 2 h in 600 μl of digestion buffer (30 μl of 10% sodium dodecyl sulphate and 570 μl of TE buffer [10 mM Tris-HC1 (pH: 8), 1 mM EDTA]).

After homogenisation, the samples were incubated in a solution of CTAB-NaCl (100 μl of 5 M NaCl and 80 μl of 10% CTAB) for 10 minutes at 65°C, and then mixed with 750 μl of chloroform-isoamyl alcohol (24:1 [vol/vol]) and centrifuged for 15 minutes at 14,000 *g *in an Eppendorf centrifuge. The aqueous phase was separated, mixed with 750 μl of phenol-chloroform/isoamyl alcohol (25:24:1 [vol/vol/vol]) and centrifuged for 15 minutes at 14,000 *g *in an Eppendorf centrifuge. The obtained aqueous phase was mixed with an equal volume of isopropanol. The samples were left at -80°C for 1 h and then centrifuged for 15 minutes at 14,000 *g*. The DNA pellet was washed up once with 70% ethanol, air dried, and dissolved in a final volume of 100 μl of TE buffer.

### PCR assay

Initially, the extracted DNA was tested for human β-globin gene to check that there were no PCR inhibitors in the samples. Primers β-GPCO (5'-ACACAACTGTGTTCACTAGC-3') and β-GPCPO (5'-GAAACCCAAGAGTCTTCTCT-3') were used to amplify a 209-bp fragment of the human β-globin gene [19]. Samples found to be negative by PCR for β-globin were retested after dilution 10-fold in distilled water. Samples shown to be β-globin positive were then examined for the presence of *U. urealyticum, U. parvum, M. hominis *and *M. genitalium*. A forward primer, My-ins (5'-GTAATACATAGGTCGCAAGCGTTATC-3'), and two reverse primers, MGSO-2-Bi (5'-CACCATCTGTCACTCTGTTAACCTC-3') and UGSO-Bi (5'-CACCACCTGTCATATTGTTAACCTC-3'), were used to amplify an approximately 520-bp region of the 16S rRNA gene of mycoplasmas and ureaplasmas [20].

The PCR mixture, which was made up to 50 μl with sterile water, contained 1 × PCR buffer [50 mM Tris-HCl (pH: 8.3), 10 mM KCl, 5.0 mM (NH4)2SO4, 2.0 mM MgCl2]; 0.5 μM My-ins; 0.25 μM (each) MGSO-2-Bi and UGSO-Bi; 0.2 mM (each) dATP, dCTP, and dGTP; 0.6 mM dUTP; 1.25 U of Go *Taq *DNA polymerase (Promega, France); and 10 μl of prepared DNA solution. PCR was performed using the Gene-Amp PCR System 9700 (Perkin Elmer Cetus) under the following conditions: an initial cycle at 95°C for 5 minutes, followed by 40 cycles of denaturation at 94°C for 30 seconds, annealing at 55°C for 30 seconds, and elongation at 72°C for 45 seconds, with a final cycle at 72°C for 7 minutes. Each run PCR included a positive control (*M. hominis *PG21) and 2 negative controls (previously negatively tested samples and distilled water). The PCR products were then subjected to hybridization assays.

### Hybridization

Amplified products of 520-bp of *U. urealyticum, U. parvum, M. hominis *and *M. genitalium *were detected by using molecular hybridization technique in a liquid phase assay (Argene Hybridowell™Universal) with species-specific internal biotinylated probes [20]:

Uure-P4-Am (5'-biotin-GGCTCGAACGAGTCGGTGT-3') specific for *U. urealyticum*,

Upar-P6-Am (5'-biotin-GTCTGCCTGAATGGGTCGGT-3') specific for *U. parvum*,

Mhom-P10-Am (5'-biotin – GACACTAGCAAACTAGAGTTAG-3') specific for *M. hominis*,

Mgen-P3-Am (5'-biotin-TCGGAGCGATCCCTTCGGT-3') specific for *M. genitalium*.

The amplified products were denatured in denaturation solution for 10 minutes and then immobilized to a microtiter plate, DNA-BIND 1 × 8 strip well plates, as described in the manufacturer's instructions. Specific-species capture probe diluted in hybridization solution was added to the wells of each microtiter plate immobilized by the denatured amplicons, and hybridization was carried out at 37°C for 30 minutes. Following hybridization, the wells were washed four times (soaking for 30 seconds) with washing solution. One hundred microliters of ready-to-use conjugate were added per well and incubated for 15 minutes at room temperature. Wells were washed as described above. One hundred microliters of substrate (Tetramethylbenzidine) were added per well and incubated for 30 minutes at room temperature in the darkness. The reaction was stopped by adding 100 μl of stop solution to each well, and the OD at 450 nm was measured.

Samples with an OD value of greater than the cut-off value (OD of negative control + 0.15) + 10% of cut-off value were considered positive, as suggested by the manufacturer.

### Statistical analysis

Semen characteristics were compared between ureaplasmas semen-positive and ureaplasmas semen-negative groups, and between mycoplasmas semen-positive and mycoplasmas semen-negative groups. Means were calculated for the separate groups for sample volume, pH, sperm concentration, vitality, rapid progressive motility, total progressive motility, normal morphology and leukocyte count for semen samples.

All the variables were initially inspected for normally distribution, and the sample volume and pH were all found to be approximately normally distributed. Therefore, standard parametric techniques (*t*-tests) were used to test significance of factors. Because sperm concentration, vitality, rapid progressive motility, total progressive motility, normal morphology and leukocyte count were not normally distributed and nonparametric techniques (Wilcoxon tests) were used to test the significance of the differences between groups regarding these factors. *P *< 0.05 was considered statistically significant.

## Results

### Frequency of genital ureaplasmas and mycoplasmas in semen samples

Of 120 semen samples, 23 (19.2%) were positive for genital ureaplasmas and 19 (15.8%) were positive for genital mycoplasmas. *U. urealyticum *was detected in 18 patients (15%). Thirteen of them had only *U. urealyticum*, and the rest consist of mixed species (1 *U. urealyticum + M. hominis + M. genitalium; *2 *U. urealyticum + M. hominis *and 2 *U. urealyticum + M. genitalium*).*U. parvum *was detected in 5 patients (4.2%). Three of them had only *U. parvum *and two had mixed species (*U. parvum + M. genitalium*).*M. hominis *was detected in 13 patients (10.8%). While nine patients had only *M. hominis*, the rest had mixed species (1 *M. Hominis + U. urealyticum + M. genitalium; *2 *M. hominis + U. urealyticum *and 1 *M. hominis + M. genitalium*).*M. genitalium *was detected in 6 patients (5%). All of them had mixture species listed above. The distribution of detected species of genital ureaplasmas and mycoplasmas from the patients is shown in Table 1.

**Table 1:** Frequency of genital mycoplasmas and ureaplasmas in semen samples of 120 infertile male patients by PCR-microtiter plate hybridization

**Species**	**Patients N = 120**
*U. urealyticum *only	13 (10.8%)
*U. parvum *only	3 (2.5%)
*M. hominis *only	9 (7.5%)
*M. genitalium *only	-
Mixed infection	8 (6.7%)
*U. urealyticum *+ *M. hominis *+ *M. genitalium*	1
*M. hominis *+ *M. genitalium*	1
*U. urealyticum *+ *M. hominis*	2
*U. parvum *+ *M. genitalium*	2
*U. urealyticum *+ *M. genitalium*	2

### Relationship between the detection of genital ureaplsmas and mycoplasmas and sperm seminological variables

The analysis of the semen specimens based on seminological variables according to the WHO criteria [18] had shown that 5.8% (7/120) were normal categories and 94.2% (113/120) were abnormal categories. The frequency of genital ureaplasmas and mycoplasmas DNA in semen samples were not significantly different between the normal and abnormal categories (*p *> 0.05).

The mean values of seminal volume, pH, sperm concentration, sperm vitality, sperm motility, sperm morphology and leukocyte count were not significantly related to the detection of genital ureaplasmas DNA in semen specimens (Table 2).

**Table 2:** Seminological variables of semen of ureaplasmas-positive and ureaplasmas- negative of 120 infertile men

	*Ureaplasma Urealyticum*			*Ureaplasma parvum*		
		
**Variable**	**Positive semen (n = 18)**	**Negative semen (n = 102)**	***P ***^# ^**value**	**Positive semen (n = 5)**	**Negative semen (n = 115)**	***P ***^# ^**value**
Volume	3.02 ± 0.33	3.41 ± 0.16	0.34*	4.38 ± 0.73	3.31 ± 0.15	0.14*
PH	7.57 ± 0.05	7.62 ± 0.03	0.58 *	7.80 ± 0.20	7.60 ± 0.03	0.19*
Sperm concentration (x10^6^/mL)	34.15 ± 8,88	50.99 ± 6.34	0.40	38.90 ± 2.70	48.87 ± 5.81	0.43
Vitality (%)	64.00 ± 3.63	61.93 ± 2.08	0.91	65.60 ± 8.22	62.04 ± 1.92	0.62
Total progressive motility (category [a + b]) (%)	31.43 ± 3.12	30.16 ± 1.34	0.72	29.00 ± 4.30	30.40 ± 1.27	0.55
Rapid progressive motility (category [a]) (%)	11,79 ± 2,49	9,67 ± 0.82	0.52	9.00 ± 3.67	10.00 ± 0.81	0.85
Morphology (Normal forms) (%)	10.07 ± 1.69	14.05 ± 1.22	0.33	12.50 ± 6.91	13.54 ± 1.10	0.52
Leukocyte count (x10^6^/mL)	0.743 ± 0.156	1.199 ± 0.279	0.053	0.348 ± 0.128	1.165 ± 0.249	0.72

*Note*: Data are means (± Standard Error)

^# ^Unless indicated, variables were tested by the Wilcoxon rank-sum test.

* Tested by *t*-test.

The sperm concentration and the percentage of normal forms of spermatozoa in the male partners of infertile couples with *M. hominis *DNA in semen specimens were significantly lower than that of the male partners without *M. hominis *DNA (14.14 × 10^6^/mL vs 52.63 × 10^6^/mL;*p *= 0.007 and 8.56% vs 13.98%. *p *= 0.03 respectively) (Table 3). The sperm concentration of spermatozoa in the male partners of infertile couples with *M. genitalium *DNA in semen specimens were significantly lower than that of the male partners without *M. genitalium *DNA (21.74 × 10^6^/mL vs 49.87 × 10^6^/mL;*p *= 0.05) (Table 3). The mean values of seminal volume, pH, vitality, motility and leukocyte count were not significantly related either to the detection of genital mycoplasmas DNA or to the detection of ureaplasmas DNA in semen specimens (Table 2, Table 3).

**Table 3:** Seminological variables of semen of mycoplasmas-positive and mycoplasmas- negative of 120 infertile men

	*Mycoplasma hominis*			*Mycoplasma genitalium*		
		
**Variable**	**Positive semen (n = 13)**	**Negative semen (n = 107)**	***P*^# ^value**	**Positive semen (n = 6)**	**Negative semen (n = 114)**	***P*^# ^value**
Volume	3.31 ± 0.37	3.35 ± 0.16	0.93*	4.32 ± 0.98	3.30 ± 0.14	0.13*
pH	7.45 ± 0.10	7.63 ± 0.03	0.07*	7.50 ± 0.00	7.61 ± 0.03	0.48*
Sperm concentration (x10^6^/mL)	14.14 ± 4.84	52.63 ± 6.10	0.007	21.74 ± 16.20	49.87 ± 5.78	0.051
Vitality (%)	57.67 ± 5.93	62.64 ± 1.96	0.33	41.00 ± 35.00	62.63 ± 1.81	0.57
Total progressive motility (category [a + b]) (%)	26.67 ± 4.41	30.68 ± 1.28	0.30	20.00 ± 20.00	30.53 ± 1.21	0.63
Rapid progressive motility (category [a]) (%)	7.78 ± 2.52	10.16 ± 0.83	0.43	7.50 ± 7.50	10.00 ± 0.79	0.71
Morphology (Normal forms) (%)	8.56 ± 3.2	13.98 ± 1.14	0.03	11.00 ± 4.00	13.55 ± 1.10	0.99
Leukocyte count (x10^6^/mL)	1.153 ± 0.529	1.128 ± 0.261	0.06	0.972 ± 0.307	1.139 ± 0.251	0.09

*Note*: Data are means (± Standard Error).

^# ^Unless indicated, variables were tested by the Wilcoxon rank-sum test.

* Tested by *t*-test.

The comparison of the sperm seminological variables between semen with mixed infections and semen without infections and between semen with mixed infections and semen without mixed infections demonstrated no significant differences in the mean values of seminal volume, pH, sperm vitality, sperm motility, sperm morphology and leukocyte count (Table 4). Only the sperm concentration in the semen specimens of infertile men with mixed infection were significantly lower than that of the semen specimens without infections (14.94 × 10^6^/mL vs 55.30 × 10^6^/mL;*p *= 0.02) (Table 4).

**Table 4:** Seminological variables of semen with mixed mycoplasmas and ureaplasmas species infections

**Variable**	**Semen with mixed infections (n = 8)**	**Semen without infections (n = 87)**	***P*^#a ^value**	**Semen without mixed infections (n = 25)**	***P***^#b ^**value**
Volume	3.74 ± 0.60	3.35 ± 0.17	0.52*	3.23 ± 0.32	0.45*
PH	7.56 ± 0.06	7.63 ± 0.03	0.55*	7.55 ± 0.08	0.95*
Sperm concentration (x10^6^/mL)	14.94 ± 7.27	55.30 ± 7.26	0.02	35.37 ± 6.96	0.064
Vitality (%)	64.50 ± 11.53	62.86 ± 2.19	0.63	59.50 ± 3.76	0.50
Total progressive motility (category [a + b]) (%)	28.75 ± 9.66	30.82 ± 1.44	0.97	28.8 ± 2.28	0.51
Rapid progressive motility (category [a]) (%)	12.50 ± 4.33	10.13 ± 0.89	0.50	8.86 ± 1.83	0.32
Morphology (Normal forms) (%)	12.25 ± 6.97	14.76 ± 1.33	0.34	9.14 ± 1.28	0.91
Leukocyte count (x10^6^/mL)	0.717 ± 0.240	1.239 ± 0.319	0.10	0.886 ± 0.284	0.83

*Note*: Data are means (± Standard Error).

^a ^Difference of seminological variables between semen with mixed infections and semen without infections.

^b ^Difference of seminological variables between semen with mixed infections and semen without mixed infections.

^# ^Unless indicated, variables were tested by the Wilcoxon rank-sum test.

* Tested by *t*-test.

### Limitations of this study

We couldn't get semen samples from fertile men, so, we have limited our comparison for seminological variables between semen from infected and non-infected infertile men with genital mycoplasmas and ureaplasmas.

## Discussion

It is estimated that 15% of male infertility is related to genital tract infection [21]. Among infectious microorganisms, *U. urealyticum *is one of the most common species [11,22]. Since 1967, the ureaplasmas have been shown as an etiology of male infertility [23], and especially when Friberg and Gnarpe [24] first demonstrated a higher frequency of ureaplasmas in the semen of men with unexplained infertility (76%) compared with fertile men (19%). Previously, *U. urealyticum *had been differentiated, into biovars 1 and 2. biovar 1 is composed of serovars 1, 3, 6, and 14, and biovar 2 is composed of serovars 2, 4, 5, and 7 to 13 [2,25]. In 1999, *U. urealyticum *biovars 1 and 2 were classified into *U. parvum *and *U. urealyticum*, respectively [26]. Most of the previous reported studies have discussed the role of ureaplasmas in male infertility without discriminating between *U. parvum *and *U. urealyticum *[9,14,15,23]. In our study, we have used the PCR-microtiter plate hybridization assay that can facilitate the identification of *U. urealyticum, U. parvum, M. hominis *and *M. genitalium *in semen specimens. Our results demonstrated that genital mycoplasmas and ureaplasmas seem to be widespread among infertile male patients, as shown respectively by the frequency of 19.2% and 15.8%. These data are comparable with those reported in previous studies [1,10,27]. *U. urealyticum *was the most prevalent species detected (15%) in this study. The frequency of *U. urealyticum *in the semen samples of male infertile patients in the literature varies from 5 to 42% [9-11,27,28]. This wide range might be explained by the diversity of detection methods used for characterizing the studied populations.

In our study, *U. parvum *was detected in 4.2% of semen samples. The frequency of this species was lower than that reported by Knox *et al*. [17] (4.2% vs 19.2%).

*M. hominis *has been associated with bacterial vaginosis, pelvic inflammatory disease, postpartum fever, and postabortal fever, as well as a number of gynaecological infections [20,29]. However, its role in non-gonoccocal urethritis (NGU) and in infertility is rarely investigated [30]. The frequency of *M. hominis*, in our study, was comparable to that reported by Andra-Rocha *et al*. [5] but higher than that reported by Rosemond *et al*. [28].

*M. genitalium *was first isolated in urethral cultures from two men with NGU in 1981 [31]. Although *M. genitalium *has been suggested as a cause of human NGU, the precise role of this mycoplasma in the etiology of NGU remains not established because of the immense difficulty in isolating it from clinical samples. More recently, however, PCR-based assays have facilitated the detection of *M. genitalium *in clinical samples [4] and a significant association has been demonstrated between *M. genitalium *and NGU [5,32]. Hitherto, *M. genitalium *has seldom been investigated in semen of infertile men. In our study, the frequency of *M. genitalium *was higher than that reported by Kjaergaard *et al*. [33] (5% vs 0.9%). This difference might be explained by the use of different methods for the detection of this bacterium. We have used PCR that is more sensitive than culture and that can facilitate the detection of *M. genitalium *in clinical samples [20].

In the present study, the frequency of the *U. urealyticum *was higher than that of *M. hominis*. *U. urealyticum *was also detected more often than *U. parvum *and *M. genitalium*. These findings were consistent with other studies [10,27,33].

In the literature, mixed species (*U.urealyticum *+ *M. hominis*) have been found in 7–14% of semen samples of infertile men [27,29]. In our study, although *M. genitalium *was not separately isolated among patients, it was detected together with *U. urealyticum + M. Hominis *in one patient, with *M. hominis *in one patient, with *U. parvum *in two patients and with *U. urealyticum *in two patients.*U. urealyticum *and *M. hominis *were observed together in two patients. Thus, eight patients (6.7%) had mixed species and our results are similar to those of previously reported studies [27,29]. These results show that the hybridization-based microtiter plate assay can be a useful method to detect mixed infection when multiple species of mycoplasmas or ureaplasmas were present in semen specimens.

Previous studies have reported that the presence of mycoplasmas and ureaplasmas in sperm specimens has no real effect on the semen quality, nor on the leukocyte count [10,29]. Recent investigations seem to show that the presence of mycoplasmas reflects a silent infection rather than infection in infertile patients [30], even though when the attachment and invasiveness towards human sperm cell has been demonstrated *in vitro *[34,35]. Reports are controversial about the effects of genital mycoplasmas and ureaplasmas infections or infection on sperm seminological variables [11,27,36,37]. We have compared semen and first void urine specimens from the 120 infertile men for the detection of genital ureaplasmas and mycoplasmas infections using in-house PCR (unpublished data). We have found a very high concordance (> 95%) and a very good agreement (K > 0.8) between the detection of genital mycoplasmas and ureaplasmas DNA in semen and corresponding first void urine specimens. Several studies have shown that nucleic acid amplification tests performed on first void urine samples are able to detect as many or more infected patients than traditional swabs from the urethra or cervix or semen [38-42]. In some cases, we have found discrepancies between the detection of genital mycoplasmas and ureplasmas DNA in semen and corresponding first void urine specimens. The presence of genital mycoplasmas and ureaplasmas DNA in first void urine samples and its absence in semen specimens may indicate an asymptomatic urethral infection. The detections of genital mycoplasmas and ureaplasmas DNA only in semen may indicate that these organisms are harboured in the epididymis or seminal vesicles.

In the present study, the comparison of the sperm seminological variables of *U. urealyticum*-positive and *U. urealyticum*-negative infertile men demonstrated no significant differences in sperm seminological variables, which confirms previous findings [27,33]. Conversely, a relationship between *U. urealyticum *and semen characteristics was observed in some literature [11,36,37]. The influence or the lack of influence of mycoplasmas and ureaplasmas on seminology may come from the capability of bacterial species to attach to spermatozoa and to affect directly via cellular interactions their vitality, motility, morphology, cellular integrity and their molecular structure or the development of protective immunity to genital infection by the host (population sensitivity to microbial agents) or other host factors.

In our study, a positive correlation was found between sperm morphology and the detection of *M. hominis *in semen samples of infertile men. Yet, a correlation was reversely observed between sperm concentration and the detection of this organism in semen samples. The sperm concentration (14.14 × 10^6^/mL) was lower than the normal reference of WHO manual (≥ 20 × 10^6^/ml) in semen of *M. hominis*-positive infertile men and higher (52.63 × 10^6^/mL) in semen of *M. hominis*-negative infertile men. The present data show that *M. hominis *may affect sperm concentration and sperm morphology of infertile men. A negative correlation was also found between sperm concentration and the detection of *M. genitalium *in semen samples of infertile men. Although, the sperm count with the presence of *M. genitalium *was within a normal range, a decrease in sperm concentration was significant. However, we have failed to demonstrate a correlation between sperm concentration and sperm morphology and the detection of genital ureaplasmas in semen samples. The comparison of the sperm seminological variables between semen with mixed infections and semen without mixed infections or without infections showed that only the sperm concentration in the male partners of infertile couples with mixed infection in semen specimens were significantly lower than that of the male partners without infections. Our findings show that the mixed infections have no additional effect on seminology.

Semen with *M. hominis *presented a higher mean of leukocytes than semen with negative *M. hominis; *this difference not was statistically significant. The detected mean (1.153 × 10^6 ^leukocytes/mL) was higher than the reference value of the WHO manual (≥ 1 × 10^6 ^leukocytes/mL). In contrast, the means of leukocyte count of the positive DNA in semen samples for *U. urealyticum, U. parvum *and *M. genitalium *were smaller than the reference value of the WHO manual. In addition, no significant difference was detected among our studied patients. These findings indicate that the presence of mycoplasmas and ureaplasmas in semen is not necessary associated with leukocytospermia, and thus, in spite of potentially pathogenic species. Our results are consistent with previous reports [10,36]. The unreliability of leukocytospermia levels to predict the presence of genital mycoplasmas and ureaplasmas, when evaluating subfertile men and the absence of leukocytospermia, does not exclude the presence of genital mycoplasmas and ureaplasmas [43,44].

## Conclusion

The results of our study demonstrate that the genital mycoplasmas and ureaplasmas seem to be widespread among male partners of infertile couples in Tunisia. The study of the comparison of the semen parameters of infertile men with and without genital ureaplasmas and mycoplasmas has not shown any significant differences, apart from the sperm concentration in the infection of *M. hominis *and *M. genitalium *and sperm morphology in the infection of *M. hominis*. Our results also indicate that PCR-microtiter plate hybridization assay method provides a rapid and effective measure to detect human genital mycoplasmas and ureaplasmas which is useful for etiological and epidemiological studies of these pathogens.

Little information was, however, available regarding the effect of mycoplasmas and ureaplasmas on the sperm quality, as well as their relationship with the leukocyte count. Therefore, it can be concluded that the screening of mycoplasmas and ureaplasmas species in routine semen analysis is not clinically relevant in our specific population. It should be restricted for men undergoing complete evaluation of infertility, genital infection and male partners from couples undergoing IVF.

## Competing interests

The author(s) declare that they have no competing interests.

## Authors' contributions

Authors R.G. carried out the PCR experiments, the analyses and interpretation of data, and drafted the manuscript. Author W.K. participated in the collection of data, PCR experiments and analysis of data. Author C.C. participated in the analysis and interpretation of sperm sminological variables. Author A.Z. participated in design and coordination of the study. Authors L.K., T.R and A.H. participated in design, data analyses, coordination and study of the manuscript.

All authors readed and approved the final manuscript.

## Pre-publication history

The pre-publication history for this paper can be accessed here:


